# Pitfalls in the Neuroimaging of Glioblastoma in the Era of Antiangiogenic and Immuno/Targeted Therapy – Detecting Illusive Disease, Defining Response

**DOI:** 10.3389/fneur.2015.00033

**Published:** 2015-02-23

**Authors:** Raymond Y. Huang, Martha R. Neagu, David A. Reardon, Patrick Y. Wen

**Affiliations:** ^1^Center of Neuro-Oncology, Dana-Farber/Brigham and Women’s Cancer Center, Boston, MA, USA

**Keywords:** glioblastoma, pseudoprogression, pseudoresponse, antiangiogenic therapy, Immunotherapy, imaging techniques

## Abstract

Glioblastoma, the most common malignant primary brain tumor in adults is a devastating diagnosis with an average survival of 14–16 months using the current standard of care treatment. The determination of treatment response and clinical decision making is based on the accuracy of radiographic assessment. Notwithstanding, challenges exist in the neuroimaging evaluation of patients undergoing treatment for malignant glioma. Differentiating treatment response from tumor progression is problematic and currently combines long-term follow-up using standard magnetic resonance imaging (MRI), with clinical status and corticosteroid-dependency assessments. In the clinical trial setting, treatment with gene therapy, vaccines, immunotherapy, and targeted biologicals similarly produces MRI changes mimicking disease progression. A neuroimaging method to clearly distinguish between pseudoprogression and tumor progression has unfortunately not been found to date. With the incorporation of antiangiogenic therapies, a further pitfall in imaging interpretation is pseudoresponse. The Macdonald criteria that correlate tumor burden with contrast-enhanced imaging proved insufficient and misleading in the context of rapid blood–brain barrier normalization following antiangiogenic treatment that is not accompanied by expected survival benefit. Even improved criteria, such as the RANO criteria, which incorporate non-enhancing disease, clinical status, and need for corticosteroid use, fall short of definitively distinguishing tumor progression, pseudoresponse, and pseudoprogression. This review focuses on advanced imaging techniques including perfusion MRI, diffusion MRI, MR spectroscopy, and new positron emission tomography imaging tracers. The relevant image analysis algorithms and interpretation methods of these promising techniques are discussed in the context of determining response and progression during treatment of glioblastoma both in the standard of care and in clinical trial context.

## Current Challenges in Post-Treatment Imaging of Glioblastoma

Glioblastoma, the most common malignant primary tumor of the central nervous system, carries a dismal prognosis with an average median survival of 14–16 months (1, 2). This has remained largely unchanged in the last decades, despite increased understanding of molecular pathogenesis and tumor microenvironment (3, 4). The current standard of care for newly diagnosed GBM consists of maximal safe resection followed by 60 Gy fractionated radiotherapy plus continuous daily temozolomide and then 6–12-month cycles of adjuvant temozolomide (5, 6). At progression, bevacizumab is the mainstay of treatment, more recently with the addition of CCNU (7).

Therapeutic strategies to date include intensified chemotherapy regimens, targeting distinct molecular pathways, inhibiting angiogenesis, and more recently immunotherapy (8). Despite these efforts, very few agents have been approved for the treatment of glioblastoma aside from temozolomide for patients with newly diagnosed GBM and bevacizumab for patients with progressive disease (PD) (5, 9). The molecular and biological complexity of GBM, its inherent adaptability and poor response to treatment, redundancy of signaling pathways, as well as the poor penetration of therapeutic agents through the blood–brain barrier (BBB) all contribute to poor progress in approval of effective therapeutics (9). A major road-block to assessment and development of effective therapeutics, however, is the lack of reliable trial endpoints (9). While overall survival (OS) is the gold standard in assessment of efficacy, progression-free survival (PFS) and response rate (RR) are valuable endpoints, highlighting the relative benefit of a given therapy and facilitating effective drug development (6, 10). Response and progression endpoints rely on magnetic resonance imaging (MRI) and are fraught with challenges including variability in image acquisition parameters, inter-rater measurement variability, difficulty in measurement of irregularly shaped tumors, and consistent interpretation of treatment-related radiographic changes: pseudoprogression secondary to radiation and chemotherapy, as well as pseudoresponse with antiangiogenic therapy (6, 9).

Current radiographic assessment of glioblastoma is based on MRI, with extent of tumor burden assessed by appearance of enhancement on contrast-enhanced T1-weighted images. This is due to local breakdown of the BBB secondary to angiogenesis in aggressive tumors (11). Response criteria developed by Macdonald et al. (12) improved on previous radiologic assessments of tumors, such as the World Health Organization response criteria, by combining bi-directional measures of enhancing tumor burden with clinical parameters, such as corticosteroid use and neurological status (13). The Macdonald criteria classify response into four categories: complete response (CR), partial response (PR; ≥50% decrease in the sum of the products of perpendicular diameters of all measurable enhancing lesions sustained for at least 4 weeks, and stable or improved clinically), stable disease (SD), and PD (≥25% increase in sum of products of perpendicular diameters of enhancing lesion or clinical deterioration) (Table 1) (12).

**Table 1:** **Current response criteria for malignant gliomas (Macdonald criteria)**.

Response	Criteria
Complete response	Requires all of the following: complete disappearance of all enhancing measurable and non-measurable disease sustained for at least 4 weeks, no new lesions, no corticosteroids, and stable or improved clinically
Partial response	Requires all of the following: ≥50% decrease compared with baseline in the sum of products of perpendicular diameters of all measurable enhancing lesions sustained for at least 4 weeks, no new lesions, stable or reduced corticosteroid dose, and stable or improved clinically
Stable disease	Requires all of the following: does not qualify for complete response, partial response, or progression; and stable clinically
Progression	Defined by any of the following: ≥25% increase in sum of the products of perpendicular diameters of enhancing lesions, any new lesion, or clinical deterioration

*Reprinted with permission from Ref. (6)*.

In 2010, in an effort to improve radiographic response criteria in an era of new biologicals and increasing need for guidelines regarding patients enrolling in clinical trials, the Response Assessment in Neuro-Oncology (RANO) Working Group proposed updated response criteria for high-grade gliomas (6) (Table 2). Increasingly, T2-weighted imaging had been incorporated into clinical practice and is particularly useful in visualizing vasogenic edema, gliosis, chemoradiation-related treatment effects, as well as evolving infiltrative and non-enhancing tumor in an era of antiangiogenic therapies that directly alter BBB permeability (14) (Table 2). Another important advance of the RANO criteria was addressing and defining pseudoresponse and pseudoprogression (6).

**Table 2:** **Summary of the proposed RANO response criteria**.

Criterion	CR	PR	SD	PD
T1 gadolinium enhancing disease	None	≥50% ↓	<50% ↓but <25% ↑	≥25% ↑^a^
T2/FLAIR	Stable or ↓	Stable or ↓	Stable or ↓	↑^a^
New lesion	None	None	None	Present^a^
Corticosteroids	None	Stable or ↓	Stable or ↓	NA^b^
Clinical status	Stable or ↑	Stable or ↑	Stable or ↑	↓^a^
Requirement for response	All	All	All	Any^a^

*RANO, response assessment in neuro-oncology; CR, complete response; PR, partial response; SD, stable disease; PD, progressive disease; FLAIR, fluid-attenuated inversion recovery; NA, not applicable*.

*^a^Progression occurs when this criterion is present*.

*^b^Increase in corticosteroids alone will not be taken into account in determining progression in the absence of persistent clinical deterioration*.

*Reprinted with permission from Ref. (6), License Number 3484960750851*.

### Pseudoprogression

The standard of care in glioblastoma treatment involves maximal safe resection followed by radiation with adjuvant temozolomide (5, 6). Within 3 months from end of radiation treatment, 20–30% of patients show increased contrast enhancement that resolves without changes in treatment on subsequent MRI scans (6). This phenomenon termed “pseudoprogression” is likely related to enhanced inflammation and disruption of the BBB caused by radiation itself, potentially enhanced by concurrent temozolomide use (6). While the pathophysiology of pseudoprogression remains to be elucidated, it seems to be part of a spectrum of radiation-related changes ranging from subacute radiographic changes to late radionecrosis (15). Pseudoprogression has also been reported in interstitial chemotherapy with carmustine-loaded polymers, which is a therapeutic option in both newly diagnosed (16–18) and progressive (19, 20) high-grade gliomas. In patients implanted with carmustine wafers, there is a high incidence (up to 90%) of cyst development near the surgical bed (21, 22) as well as a transient increase in contrast enhancement and peri-cavity edema within the first 2 months after wafer placement (23).

Failure to recognize pseudoprogression may lead to premature discontinuation of effective adjuvant temozolomide chemotherapy and inappropriate inclusion of these patients into trials for progressive/recurrent glioma, resulting in falsely elevated RRs and PFS (6). The RANO criteria attempt to address this problem by excluding patients who “progress” during the first 12 weeks post-chemoradiation from entry into new clinical trials unless the progression is largely outside the radiation field or if there is pathologic conformation of progressive/recurrent tumor (6). Despite these advances, pseudoprogression remains a significant diagnostic challenge, and this review will discuss the advanced imaging techniques that are currently being evaluated in differentiating pseudoprogression from true progression of glioblastoma.

## Advanced Imaging Techniques for Evaluation of Treatment Response

### Differentiating pseudoprogression from tumor progression

#### Magnetic resonance perfusion imaging

The hemodynamic characteristics of brain tumors and radiation necrosis can be estimated non-invasively using perfusion imaging techniques. Three magnetic resonance perfusion techniques are increasingly available on clinical MRI scanners: dynamic susceptibility contrast (DSC)-MRI, dynamic contrast enhanced (DCE)-MRI, and arterial spin labeling (ASL).

#### Dynamic susceptibility contrast-magnetic resonance imaging

**Figure 1:**
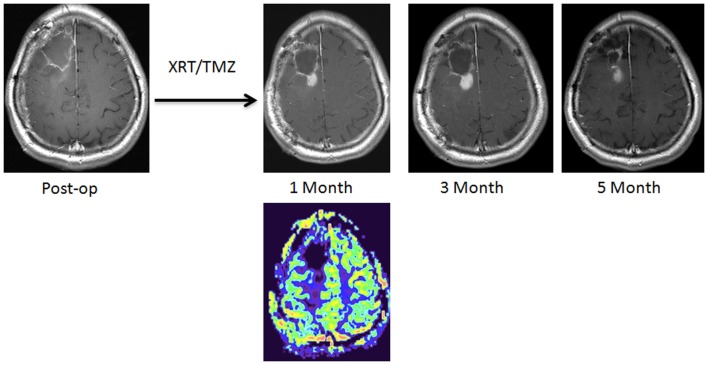
**A new enhancing lesion appeared around the resection cavity 1 month following completion of chemoradiation, without evidence of elevated rCBV on DSC-MRI**. The lesion continued to grow during the next 2 months but eventually decreased in size, consistent with pseudoprogression.

Dynamic susceptibility contrast-magnetic resonance imaging measures the signal intensity change related to T2/T2* relaxation during a first-pass bolus of paramagnetic contrast agent (24, 25). Quantitative parameters derived from the time–intensity curve using normal brain as reference are used to depict pathological alterations. These include relative cerebral blood volume (rCBV), the most commonly studied parameter in DSC-MRI for characterization brain tumor (Figure 1), as well as relative peak height (rPH) and percentage of signal intensity recovery (PSR) (26). These parameters can be normalized or standardized using normal gray and white matter for easier comparison between studies and patients (27, 28).

While a number of studies have applied DSC-MRI methods to distinguish pseudoprogression from tumor progression in glioblastoma, a wide range of sensitivity and specificity have been reported (29–34). These variations can result from small sample sizes in some of these studies as well as from differences in acquisition protocols, analytic techniques, and reference standards among the studies. Using histopathology from 57 patients as a reference standard, Barajas et al. demonstrated that rPH and rCBV were significantly greater in progressive/recurrent tumor as compared to radiation necrosis, while PSR values were significantly lower in patients with recurrent tumor (30). rPH also appears to be the best predictor of recurrent tumor compared to rCBV and PSR in this study, since the latter two parameters have significant overlaps between tumor tissue and radiation necrosis. Similarly, Hu et al. examined 40 stereotactic specimens from 13 patients and compared them with preoperative rCBV (31). With an rCBV threshold of 0.71, pseudoprogression can be differentiated from progressive/recurrent tumor with a sensitivity of 91.7% and a specificity of 100% in this small series.

The role of histopathology as the standard reference for assessment of tumor progression versus radiation necrosis is increasingly being challenged; in addition to sampling error and reader variability, post-treatment tissues often contain both viable tumor and necrotic tissues making it difficult for all-or-none diagnoses. On the other hand, imaging approaches that take into account whole-tumor heterogeneity can mitigate this problem. Hu et al. developed the concept of MRI-fractional tumor burden (pMRI-FTB) and demonstrated that this parameter correlated with the relative histologic fraction of viable tumor and was also predictive of OS (35). Analyzing perfusion maps from 79 patients with glioblastoma, Baek et al. demonstrated that histogram analysis of whole-tumor rCBV can help differentiate pseudoprogression from tumor progression with sensitivity of 85.7% and a specificity of 89.2% (34).

Perfusion maps before and after chemoradiation therapy can be analyzed simultaneously as parametric response maps (34). A decrease in rCBV and rCBF on parametric response maps, counter-intuitively, is more often observed with progressive/recurrent tumor (36). Cao et al. also reported that a decrease in fractional tumor volume with low rCBV 1 week following radiation was predictive of improved survival in 23 patients with high-grade gliomas (37). From the same study, it appears that the timing of rCBV measurement following radiation is important, since a decrease in the fractional high-CBV tumor volume in the third week versus in the first week following radiation was also predictive of a longer survival outcome. Mangla et al. have shown in 36 glioblastoma patients that an increased in percentage change of rCBV (> 5%) after radiation and temozolomide was predictive of 1-year survival with a sensitivity of 90% and a specificity of 60% (38).

**Figure 2:**
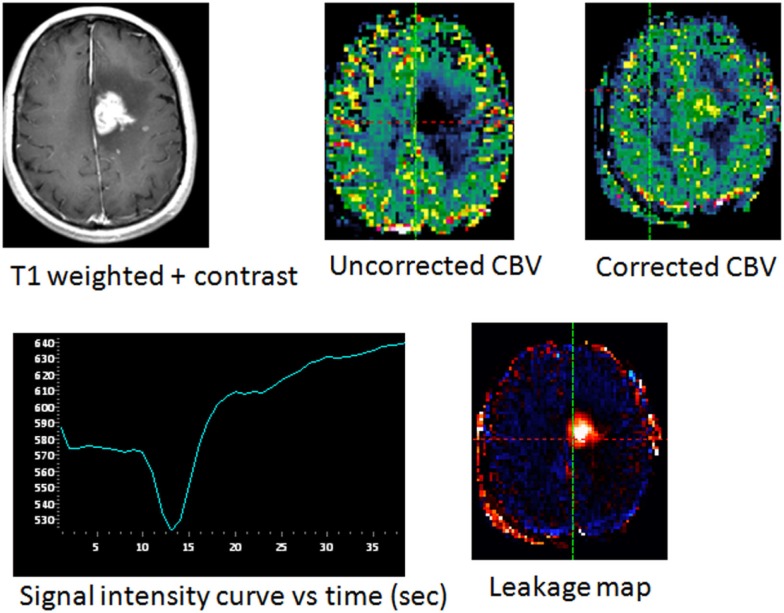
**New enhancing area in a patient with glioblastoma following chemoradiation treatment, with pathologically confirmed tumor progression**. The uncorrected CBV map showed an apparently lower blood volume relative to normal brain. Presence of significant leakage is seen within the enhancing lesion as indicated by signal intensity curve and leakage map. Following leakage correction, elevated cerebral blood volume in the enhancing region is confirmed.

While DSC-MRI has several advantages as a choice of perfusion imaging technique including ease of implementation, rapid acquisition, and an optimized signal-to-noise level, there are a number of technical limitations. Due to its sensitivity to susceptibility, the signal-to-noise level of DSC-MRI can be significantly reduced in anatomical areas near the bone or air interface, as well as near sites with significant blood products. The accuracy of rCBV can also be affected by the presence of BBB disruption resulting in T1-weighted leakage and T2/T2*-residual effects. The effect from contrast leakage can lead to either overestimation or underestimation of rCBV in tumors (39) (Figure 2). Several methods can be implemented to minimize the effect of contrast leakage permeability on rCBV calculation, including contrast preloading (40), dual-echo acquisition (41), and modeling of transvascular transfer constant (37, 42–45). Finally, blood-pool contrast agents, such as ferumoxytol, can reduce leakage effect; in patients with glioblastoma following chemoradiation, Gahramanov et al. demonstrated that rCBV calculated from DSC-MRI perfusion acquisition using ferumoxytol is predictive of OS without the need of leakage correction (46).

#### Dynamic contrast enhanced-magnetic resonance imaging

Dynamic contrast enhanced-magnetic resonance imaging techniques can characterize vascular permeability by quantifying movement of paramagnetic contrast agents crossing the BBB using pharmacokinetic models (47–49). The most widely studied variables derived from DCE-MRI in brain tumor imaging are Ktrans (transfer coefficient between the intra- and extravascular spaces), Ve (extravascular, extracellular space), and Kep (transfer constant from the extracellular, extravascular space into the plasma) (48, 49). Compared to DSC-MRI, DCE-MRI is relatively immune to susceptibility artifact and can more accurately account for contrast agent leakage effect in the calculation of cerebral blood volume. With T1-weighted image acquisitions, DCE-MRI derived perfusions maps also have greater signal-to-noise ratio and spatial resolution, although there is a need for longer imaging acquisition time.

**Figure 3:**
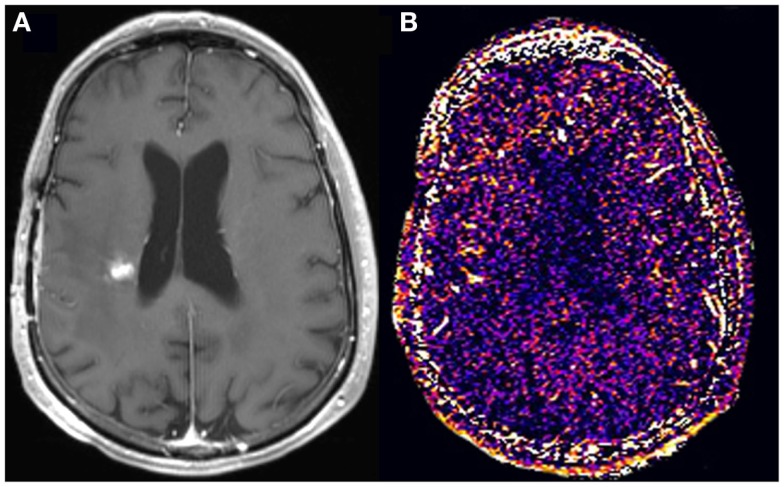
**A new enhancing lesion appeared adjacent to the resection cavity 3 months following completion of chemoradiation (A), without evidence of elevated kTrans (B) on DCE-MRI**. The lesion remained unchanged in size for the subsequent 4 months, consistent with pseudoprogression.

Several studies have applied DCE-MRI to differentiate tumor progression from radiation necrosis (Figure 3). Larsen et al. reported nearly 100% sensitivity and specificity using calculated CBV, comparable to those determined by FDG-positron emission tomography (PET) on the same patients (50). Bisdas et al. demonstrated significant greater Ktrans in progressive/recurrent tumor lesions as compared to the radiation-induced necrotic sites (*P* ≤ 0.0184). A Ktrans cutoff value higher than 0.19 showed 100% sensitivity and 83% specificity for detecting progressive/recurrent gliomas (51).

Despite the advantages of DCE-MRI, the pharmacokinetic models for calculation of physiological parameters are typically complex and require several assumptions, leading to difficulty in standardization. Non-model-based methods are easier to implement and the resulting semiquantitative parameters, while not physiologic, are more reproducible. Narang et al. assessed non-model based parameters initial area under the signal intensity–time curve (iAUC) and maximum slope of enhancement in initial vascular phase (MSIVP) to help differentiate progressive/recurrent glioblastoma from radiation necrosis in 36 patients with glioblastoma (52). Significantly higher MSIVP and iAUC (at 60 and 120 s) were observed in the progressive/recurrent tumor group, with MSIVP being the better single predictor with high sensitivity (95%) and specificity (78%).

In a larger retrospective cohort of 169 patients with pathologically or clinicoradiologically diagnosed progressive/recurrent glioblastoma (*n* = 87) or radiation necrosis (*n* = 82), Kim et al. demonstrated the addition of either DSC-MRI or DCE-MRI to contrast-enhanced T1-weighted and diffusion-weighted images improved prediction of progressive/recurrent tumor (53). However, there was no significant difference between DSC-MRI and DEC-MRI in the degree of improvement for diagnostic accuracy.

#### Arterial spin label MR perfusion

**Figure 4:**
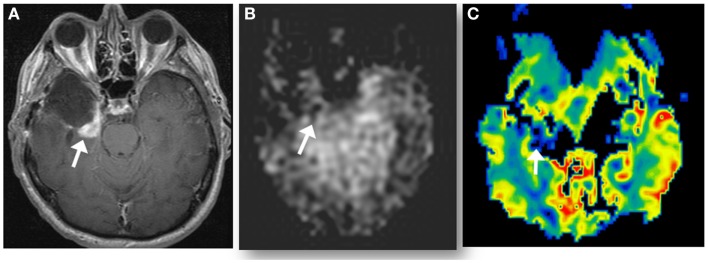
**A new enhancing lesion appeared along medial margin of right temporal resection cavity 2 months following completion of chemoradiation (A), without evidence of neither elevated CBF on ASL perfusion (B), nor elevated rCBV (C) on DSC-MRI**. The lesion was confirmed as pseudoprogression on subsequent imaging.

Arterial spin label (ASL) MR perfusion imaging estimates cerebral blood flow (CBF) by tagging endogenous blood as a flow tracer without the need of injecting exogenous contrast (54, 55). Although ASL is limited by lower signal intensity-to-noise ratio and longer acquisition time compared to DCE-MRI and DSC-MRI, the major advantage of ASL technique is its application in patients with insufficient renal excretory function and the ability to repeat ASL acquisitions during a single study. This technique has been applied to imaging of glioma, and the blood flow measurement correlates with histologic grades (56, 57). Choi et al. retrospectively evaluated the added value of ASL to DSC-MRI in 177 consecutive patients with glioblastoma following standard chemoradiation therapy (58) (Figure 4). Among the 62 patients who developed contrast-enhancing lesions, ASL grading is an independent predictor of early tumor progression and improves diagnostic accuracy when interpreted qualitatively in conjunction with DSC-MRI.

The advantages and disadvantages of the three MR perfusion techniques discussed in this review are summarized in Table 3. The clinical value of perfusion imaging has been increasingly recognized in neuro-oncology centers as recently demonstrated by Geer et al. in their analysis of the contribution of perfusion imaging to clinical decision making (59). While the acquisition and analytic algorithms have improved significantly over the last decade, for perfusion to be incorporated into standard response criteria in clinical trials and routine clinical practice, significant improvements are still needed in standardizing perfusion protocols in order to increase diagnostic accuracy and reproducibility.

**Table 3:** **Summary of MR perfusion imaging techniques**.

Perfusion technique	Advantages	Disadvantages
Dynamic susceptibility contrast (DSC)	Short imaging time	Prone to artifacts from bone, metal and air
	More widely available	
		Lower spatial resolution
		Need leakage correction
Dynamic contrast enhanced (DCE)	Higher spatial resolution	Longer imaging time
	Estimate vascular permeability	Require pharmacokinetic modeling
Arterial spin label (ASL)	Quantifies blood flow	Longer imaging time
	Does not need contrast	Lower signal-to-noise ratio

#### Magnetic resonance diffusion imaging

Magnetic resonance diffusion imaging can non-invasively examine tissue by probing microscopic water motion to indirectly assess cell density and architecture. When applied to brain tumors, diffusion imaging can assist in differentiating tumor type (60–64), as well as predicting tumor grade (65–68) and estimating prognosis (67). Most current clinical applications of diffusion imaging is performed with diffusion weighting factor b near 1000 s/mm^2^ where the diffusion signal decay is approximately mono-exponential. This single exponential constant, or apparent diffusion coefficient (ADC), can be readily calculated for each voxel and represented as a magnitude map. In brain tumor imaging, ADC has been shown to inversely correlate with tumor cell density (64, 65, 69). With increased cellularity, ADC values tend to be lower for high-grade glial tumors, likely due to restricted water motion in the midst of tightly packed tumor cells (65). On the other hand, peritumoral edema is characterized by high ADC values (60, 70). Other pathological conditions can also result in alterations in ADC values including ischemia, infection or inflammation. Thus, diffusion-weighted imaging is often interpreted alongside other MR sequences to increase diagnostic specificity. One important cause of new or increased enhancement following chemoradiation is due to postsurgical infarction (71). Thus, examining diffusion-weighted images of immediate postoperative MRI is important in making this diagnosis.

**Figure 5:**
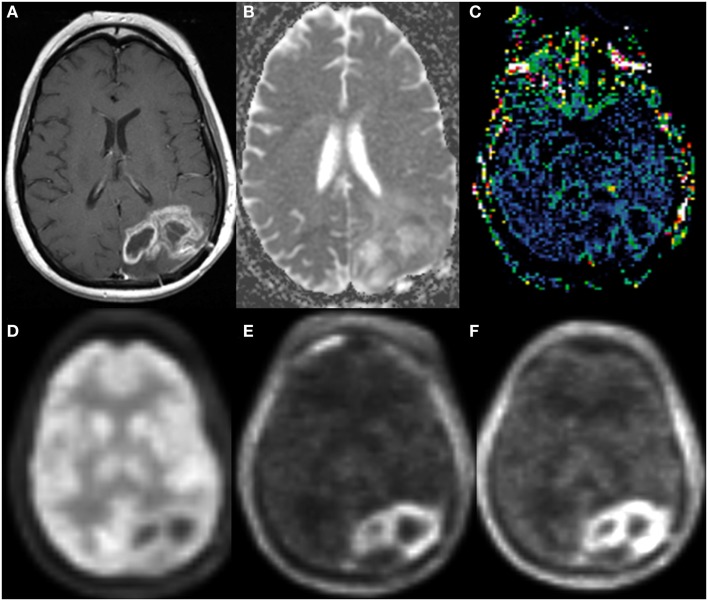
**Enlarging left parieto-occipital lobe enhancement 4 months following chemoradiation therapy (A)**. The enhancing region demonstrated low ADC **(B)** on DWI, mildly elevated rCBV **(C)** on DSC-MRI. FDG-PET **(D)** of the same lesion had less conspicuous lesion to background uptake compared to FLT-PET **(E)** and FET-PET **(F)**. Subsequent resection confirmed progressive/recurrent glioblastoma.

Several prior studies have demonstrated lower ADC values with respect to normal brain tissues in patients who received radiation and chemotherapy and had subsequently confirmed tumor progression (72–74) (Figure 5). Furthermore, an increase in tumor ADC values following therapy compared to pre-treatment ADC has been shown to be predictive of favorable response (75, 76). While the results from these studies with small patient sample size support the value of diffusion MRI in differentiating pseudoprogression from progressive/recurrent tumor, there are several limitations that need to be considered when including this technique as part of diagnostic algorithm. First, variations in MRI equipment and acquisition parameters can result in differences in calculated ADC values, and even ratio values using normal appearing brain as a reference can produce inconsistent results. This could be one reason for a lack of consistent threshold values allowing for differentiating tumor progression from necrosis. Second, ADC values within a single tumor are often heterogeneous, likely reflecting a mixture of viable and necrotic tumor tissue as mentioned earlier. Thus, ADC analyses using mean or median in tumor volume of interest may not be sensitive to spatial heterogeneity, resulting in inaccurate diagnosis of tumor progression. Histogram-based methods have been developed to characterize relative mixtures of ADC values and tested as predictors of patient outcome (77, 78). While promising, implementation of this approach in routine practice remains challenging due to a requirement for tumor volume segmentation but can be facilitated with automated or semi-automated volume segmentation techniques.

#### Magnetic resonance spectroscopy

Magnetic resonance spectroscopy (MRS) can non-invasively measure concentrations of tissue metabolites and has shown promising applications in evaluating brain tumors including their diagnosis, grading, pre-therapy planning, and post-therapy assessment (79). MRS data can be acquired using single-voxel technique by manually defining regions-of-interest within brain lesions, and altered levels of several known metabolites including *N*-acetyl aspartate (NAA), choline, creatine, and lactate provide a basis for distinguishing suspected progressive/recurrent tumor from treatment-related changes (80–83). While higher choline to creatine and choline to NAA ratios were observed in tumor progression compared to normal appearing brain or treatment necrosis, classification of tissues containing mixtures of tumor and necrosis using single-voxel techniques can be challenging. A multi-voxel acquisition approach (chemical shift imaging) can account for spatial heterogeneity in tissues and appears to improve diagnostic accuracy for detecting tumor (84–89). A recent meta-analysis reported that the diagnostic performance in differentiating glioma progression from radiation necrosis using choline to NAA ratio has sensitivity and specificity of 0.88 and 0.86, respectively (90). In addition, biochemical changes during post-treatment necrosis appear to have temporal variability, including decreased NAA concentrations over time and a transient increase in choline following radiation therapy (80, 82, 86, 91, 92), suggesting that a longitudinal evaluation using MRS may provide greater specificity.

While the diagnostic value of MRS in distinguishing progressive/recurrent tumor from treatment-induced necrosis remains to be validated, a number of challenges need to be addressed. First, MRS acquisitions using either single- or multi-voxel methods require manual input for selecting region-of-interest and placement of saturation bands, thereby introducing variability dependent on user experiences. The relative lower spatial resolution of MRS compared to conventional imaging sequences can introduce uncertainty during spectral acquisitions due to inclusion of non-lesional tissues, such as normal brain, surgical cavity, or subarachnoid spaces, requiring expert review of conventional sequences when interpreting MRS findings. Finally, MR equipment, pulse sequences, parameters, and data post-processing methods can also affect measurement reproducibility across treatment sites. These technical challenges are important to overcome for standardization and implementation of this promising technique.

#### Positron emission tomography

Compared to normal brain tissues, tumors often carry greater metabolic activity, which can be detected by PET imaging as increased uptake of 18*F*-fluorodeoxyglucose (18*F*-FDG), a radio-labeled glucose analog (93). For primary brain neoplasms, the degree of 18*F*-FDG uptake on PET has been correlated with both tumor grade (94, 95) and patient survival (96–102).

18*F*-Fluorodeoxyglucose PET has also become a valuable tool for assessing treatment response in a number of human cancers (103). Several previous studies have examined the utility of FDG-PET in distinguishing radiation necrosis from tumor following radiation treatment, with a broad range of sensitivities and specificities reported (104–108). However, most of the patients included in these studies developed lesions with new or increased enhancement on MRI more than 3 months after therapy, while the majority of patients with pseudoprogression experience imaging findings within the first 3 months after chemoradiation. Thus, the results from the application of FDG-PET imaging techniques in delayed post-radiation necrosis and tumor progression may not be directly translated to its ability to distinguish between the subacute post-radiation changes (i.e., pseudoprogression) and true tumor progression. Furthermore, the use of FDG-PET in assessment of tumor progression is limited by a number of factors. First, due to the relative intrinsic high metabolism in normal brain cortex, measurement of FDG uptake within lesions near gray matter can be difficult. While delayed phase FDG-PET imaging may improve discrimination between glioma and normal gray matter, evidence supporting its use remains preliminary (109, 110). Second, the sensitivity of FDG-PET in determining tumor progression can be limited by the intrinsic changes of recurrent tumor affecting FDG-PET uptake; while high-grade gliomas tend to be hypermetabolic on FDG-PET (111), the level of FDG uptake of progressive/recurrent tumor may differ from that of the original tumor. Third, radiation necrosis may be associated with inflammatory processes and increased glucose metabolism (112), making elevation of FDG uptake less specific in this setting. Finally, the resolution of PET imaging is currently limited to 5 mm. While co-registration with CT and PET on dedicated scanners can improve accuracy of lesion localization, detection and assessment of small lesions in the setting of early recurrence remains difficult. Despite these limitations, FDG-PET imaging has become widely available in major cancer centers, making this an important diagnostic tool for detecting tumor recurrence when combined with advanced MR imaging.

#### Amino acid PET

The short-coming of the relatively low tumor-to-background FDG uptake prompts investigations of other tumor-sensitive radiotracers with intrinsically low accumulation by normal brain tissues. In malignant brain tumors, higher proliferative activities in neoplastic cells result in increased amino acid transport (113–116), providing a basis for using radio-labeled amino acids as target for brain tumor in PET imaging. Due to relative slow uptake of amino acid in normal brain, amino acid radiotracer has the important advantage of high lesion-to-background uptake for imaging of brain tumors (Figure 5).

l-Methyl-11*C*-methionine (11*C*-MET) is the most widely characterized amino acid radiotracer in imaging of brain tumors (117–120). Compared to FDG, 11*C*-MET PET is superior in detecting tumor progression (121, 122), even in cases where there is normal or low FDG uptake by tumors (123). Other C11 based radiotracers also have shown similar promising results, including l-1-[11*C*]-tyrosine (11*C*-TYR) (124–126). However, due to the relatively short half-life of 11C, clinical application of 11*C*-MET and 11*C*-TYR require on-site cyclotrons and their current availability remains quite limited.

Amino tracers with longer half-life radiolabel include 3′-fluoro-3′-deoxy-l-thymidine (18*F*-FLT) (127–130), *O*-2-18 *F*-fluoroethyl-l-tyrosine (18F–FET) (131–135), and 3,4-dihydroxy-6-[18*F*]-fluoro-l-phenylalanine (18*F*-FDOPA) (136–141). These tracers all share the same features of lower normal cortical tracer uptake and facilitate clinical implementation due to the longer half-life of F18. Furthermore, kinetic modeling of radiotracer uptake may provide additional specific markers that can distinguish between tracer uptake due to BBB leakage, such as the case of radiation necrosis, and tracer accumulation due to increased active transport in growing tumors (129, 130). While preliminary results identify these radiotracers as early markers of treatment response and survival, (141–144), their roles in distinguishing treatment-related changes from true progressive/recurrent tumor remain to be validated in larger prospective trials.

#### Antiangiogenic therapy and pseudoresponse

Direct correlation of enhancing disease burden with glioma progression is particularly challenging in the context of antiangiogenic therapies targeting vascular endothelial factor (VEGF), such as bevacizumab, a recombinant humanized monoclonal antibody to VEGF-A, or the VEGF receptor such as cediranib, a pan-VEGF receptor tyrosine kinase inhibitor (6, 145). Through normalization of leaky tumor blood vessels, these agents can cause reduction in enhancement within 1–2 days after administration, with a radiographic response in 25–60% of patients (146). This impressive radiographic response unfortunately does not translate into increased survival. It is thought that this rapid radiographic response represents a direct action on blood vessel permeability rather than a true anti-tumor effect; a phenomenon termed “pseudoresponse” (6, 147). The RANO criteria address this issue by requiring a radiographic response to persist for more than 4 weeks in order to be considered a true response (6, 147). A further confounder in radiographic assessment of response is the tendency for antiangiogenic agents to promote progression of non-enhancing disease by selecting for an invasive tumor phenotype capable of co-opting existing blood vessels and no longer relying on angiogenesis (6, 147). T2-weighted or FLAIR images best represent infiltrative disease. The radiographic appearance of infiltrative tumor is often subtle and diverse, including evidence of mass effect and invasion of the cortical ribbon. Given the radiographic variability of non-enhancing infiltrative disease, the RANO group concluded current technologies fell short of providing objective measures of infiltrative tumor progression (6). Since progression of infiltrative tumor often causes clinical deterioration, the RANO criteria include the patient’s clinical status in assessment of progressive non-enhancing tumor (6). Clearly, this is a suboptimal imaging surrogate and highlights the acute need for superior technologies in assessment of non-enhancing tumor progression. Here, we review promising advanced imaging modalities for the assessment of tumor burden in the context of antiangiogenic therapy.

### Detecting tumor in antiangiogenic therapy

#### T1 subtraction map

Due to their effect on vascular permeability, antiangiogenic agents result in a dramatic reduction in contrast enhancement within tumor on T1-weighted images soon after initiation of therapy that is unrelated to anti-tumor effect. While the relative reduction in enhancement can be interpreted as non-enhancement by visual analyses, quantitative methods using voxel-to-voxel image subtraction between T1-weighted images before and after contrast administration may detect subtle residual enhancement and therefore provide a more accurate and reproducible assessment of true tumor extent in the context of antiangiogenic treatment (148, 149). Recently, Ellingson et al. analyzed 160 patients from the phase II randomized clinical trial (AVF3708g, BRAIN trial) in patients with glioblastoma treated with bevacizumab or bevacizumab and irinotecan using a T1 subtraction method (150). There was significantly improved visualization and quantification of tumor volume in post-treatment patients and calculated brain volume from subtracted images correlated with both PFS and OS better than those from un-subtracted post-contrast images. This method can be readily incorporated into clinical practice since pre and post-contrast T1-weighted imaging are usually part of standard protocol in brain tumor imaging, although ease-of-use post-processing software for co-registration, normalization, and subtraction are necessary. Additional technical challenges also include the need of standardizing image acquisition to minimize inter-subject and intra-subject variability.

#### T2 mapping

**Figure 6:**
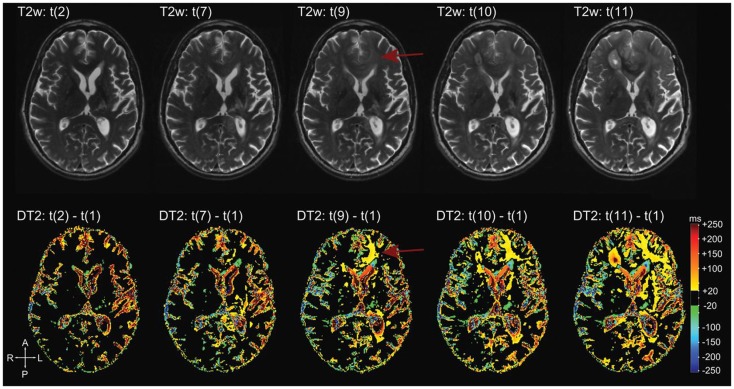
**Differential quantitative T2 maps**. Reprinted with permission from Ref. (151), License Number 3520001314812. More apparent changes on the differential T2 map (bottom row) in the left frontal lobe (arrows) compared to T2-weighted images (top row). These changes are hardly visible on conventional T2-weighted images (arrows).

While evaluation of T2/FLAIR disease in the setting of antiangiogenic therapy has been incorporated into the RANO criteria and increasingly adopted in recent clinical trials, the assessment is based on qualitative inspection without any objective guidelines. This is in part due to difficulties measuring T2/FLAIR disease with consistency and the lack of specificity for tumor tissues versus other cause of T2/FLAIR signal abnormality including edema, necrosis, and gliosis. T2 mapping is an imaging technique that quantifies T2 relaxation for each voxel using the effective echo times from two echoes acquired during a fast-spin echo preparation. Using this method, Ellingson et al. was able to perform direct voxel-to-voxel subtraction of quantitative maps before and after bevacizumab treatment in patients with glioblastoma (151). The resulting maps allowed visualization and quantification of voxel-wise T2 changes resulting from anti-VEGF therapy (Figure 6). There was a significant decrease in T2 relaxation time within pre-treatment T2 abnormal regions following treatment, and an elevated residual, post-treatment; in addition, median T2 was predictive of both PFS and OS. Hattingen et al. extended the application of this technique to generate longitudinal differential T2 maps using the first post-treatment T2 map as a reference, and demonstrated non-enhancing tumor progression more clearly than conventional T2-weighted imaging (152). While the utility of T2 mapping techniques needs to be validated with further studies, this technique is promising both as an early post-treatment predictor and as a more sensitive marker of non-enhancing tumor progression in antiangiogenic therapy.

#### Diffusion MRI in antiangiogenic therapy

Unlike contrast-enhanced T1-weighted imaging, diffusion imaging based on ADC analyses is relatively unaffected by alterations in vascular permeability during antiangiogenic therapy (153). This advantage makes it a potentially more accurate technique in assessing the extent of tumor in this treatment setting.

A number of studies have evaluated the predictive value of pre-treatment ADC values for both treatment response and survival outcome. Due to heterogeneity of ADC values within tumor regions, whole-tumor histogram analyses have been increasingly utilized for evaluating the effect of different ADC subcomponents that have different prognostic or predictive values. Pope et al. applied histogram analyses of ADC values within contrast-enhancing progressive/recurrent glioblastoma before bevacizumab treatment and demonstrated that the mean value of the lower component of a two-Gaussian histogram fitting is a predictor of PFS (154). This result was subsequently validated using imaging data from a multicenter trial of patients with progressive/recurrent glioblastoma treated with bevacizumab with or without irinotecan, and the pre-treatment lower ADC component of enhancing regions was associated with OS (155). The pre-treatment ADC histogram of non-enhancing T2/FLAIR in 91 patients with progressive/recurrent glioblastoma also has been characterized using a four-component fitting model, and the resultant low to middle peak ratio was shown to be a predictor of OS independent of both the extent of the enhancing region and tumor size (156).

Treatment-induced changes in ADC obtained by comparing pre- and early post-treatment measurements have also been tested as an imaging marker of treatment outcome. Nowosielski et al. examined the skewness, or degree of asymmetry, of ADC histograms in patients with progressive/recurrent glioblastoma and showed that patients with increasing skewness (*n* = 11) following bevacizumab/irinotecan therapy had significantly shorter PFS than did patients with decreasing or stable skewness (157). While histogram-based approaches can analyze the relative proportion of individual ADC subtypes, regional changes before and after therapy cannot be captured. Using functional diffusion map (fDM) methods by voxel-wise subtraction of pre- and post-treatment ADC maps, precise magnitude of change in ADC at all tumor locations can be studied. Ellingson et al. (158, 159) applied a graded fDM using multiple thresholds of ADC change to assess antiangiogenic therapy in progressive/recurrent glioblastoma and showed that the volume of decreased ADC values between 0.25 and 0.40 μm^2^/ms in both enhancing and non-enhancing regions is associated with OS.

Change in ADC can also be followed longitudinally by serial MRI. Using percentage change of low ADC volume over time, Gerstner demonstrated progressive increase of percent volume with low ADC volume within non-enhancing regions following cediranib therapy to correlate with infiltrative tumor progression (160). Similarly, Jain et al. compared mean ADC within contrast-enhancing and non-enhancing volumes and determined that patients with PD showed a sequential increase in the negative percent change of ADC values following bevacizumab therapy (161). While ADC values can correlate with tumor growth in serial imaging, developing automated methods of voxel-wise subtraction is important for real-time adoption of this method for use in prospective clinical trials.

#### High *b*-value MR diffusion imaging

In most commonly used clinical MR scanners, the *b* value of diffusion gradient is usually 1000 s/mm^2^. When the *b* value increases to beyond 3000 s/mm^2^, diffusion signal decay is no longer mono-exponential, and analysis of higher range *b* values potentially can result in greater imaging contrast between different tissue types. Applying high *b*-value diffusion imaging to characterize brain tumors, Seo et al. demonstrated that the degree of ADC decrease was greater in tumors compared with normal brain tissue (162). Using histogram analysis of ADC maps based on entire tumor volume, Kang et al. demonstrated that the histogram parameters derived from high *b* values performed better diagnostically than those from standard *b* values in differentiating high- from low-grade gliomas. ADC values decreased when the *b* value was increased from 1000 to 3000 s/mm^2^, and a greater decrease was observed with higher tumor grades (163). In 4 of 10 patients with progressive/recurrent glioma treated with bevacizumab, high *b*-value diffusion imaging identified pseudoresponse at earlier times compared to both the Macdonald and RANO criteria (164).

#### MR perfusion imaging

The ability of perfusion imaging techniques to measure blood flow dynamics *in vivo* makes them potentially useful tools not only for understanding the effect and mechanism of antiangiogenic therapy, but also for providing prognostic or predictive information important for patient selection and treatment decisions. While mechanisms of action of antiangiogenic agents are not fully understood, early decrease in Ktrans, an DCE-MR marker of vascular permeability (49), can be detected at day 1 after a single dose of cediranib in patients with progressive/recurrent glioblastoma, and the decrease has been shown to be associated with improved PFS and OS (146, 165). The improved blood flow was also associated with tumor oxygenation (166). These observations support the theory of “vascular normalization” as the mechanism of action (167), and a “vascular normalization index” (VNI) combining Ktrans and circulating collagen IV were subsequently proposed as a marker to predict survival (165). A similar VNI parameter can be obtained by comparing pre-treatment and 1-day post-treatment DSC-MRI using a single double-echo acquisition. This new VNI parameter combining changes in tumor CBV and an apparent transfer constant (Ka) using a leakage correction method was predictive of PFS and OS in 30 patients with progressive/recurrent glioblastomas enrolled in a phase II clinical trial of an oral pan-VEGF receptor tyrosine kinase inhibitor (45).

Using DSC-MRI, Essock-Burns examined 35 patients with newly diagnosed GBM who received temozolomide chemoradiotherapy with enzastaurin, an oral PKC isoform kinase inhibitor, after surgical resection (168). Responders at 6 months showed an increased percent recovery (PR) between baseline and 2 months into therapy, indicating improved permeability, whereas non-responders at 6 months showed significantly increased peak height (PH), a marker of microvascular density similar to CBV (169) between baseline and 1 month. Using standardized rCBV, a consistent intensity scale regardless of MR scanner model or field strength, Schmainda et al. examined the prognostic values of DSC-MRI findings in 36 patients with progressive/recurrent high-grade glioma 60 days before and 20–60 days after starting bevacizumab and reported longer OS if pre- or post-treatment standardized rCBV is less than 4400 (170). The use of standardized perfusion parameters can reduce variability when comparing findings from different subjects, scanners, or using different acquisition techniques, but the reported threshold values require validation in larger clinical data sets.

#### Evaluating treatment response to immunotherapy

A newer challenge is the differentiation of pseudoprogression from true progression in patients who receive immunotherapy. The FDA approval of two recent immunotherapy approaches, Sipuleucel-T (APC8015) for prostate cancer and ipilimumab for melanoma, as well as ongoing trials showing efficacy of immunotherapy against challenging cancers, heralds a new era of cancer treatment (171, 172). Ongoing immunotherapy trials in glioblastoma hold great promise for improved outcomes in this devastating illness (171, 172). Since the goal of immunotherapy is to harness the patient’s immune system to fight cancer, inflammation in the tumor bed is expected much more than with cytotoxic chemotherapies. Experience in melanoma trials has shown that while tumor regression is often low (only 10% of patients), many patients can have prolonged periods of stability (173). This would potentially go undetected using RR as an endpoint (173–175). In addition, some tumors may develop new lesions or transiently increased size before eventually becoming smaller. To re-define response in the context of immunotherapy, assess efficacy of treatment in clinical trials, prevent patients from discontinuing a potentially beneficial treatment, as well as to ensure that patients do not remain on a potentially harmful, ineffective treatment, Wolchuk et al. proposed a new set of response criteria termed immune-related response criteria (irRC) for melanoma (173). The irRC allow for patients tolerating therapy to stay on treatment beyond initial progression for another 4 weeks and define disease progression as an increase in the measurement of overall tumor burden rather than the appearance of new lesions (173, 176).

**Figure 7:**
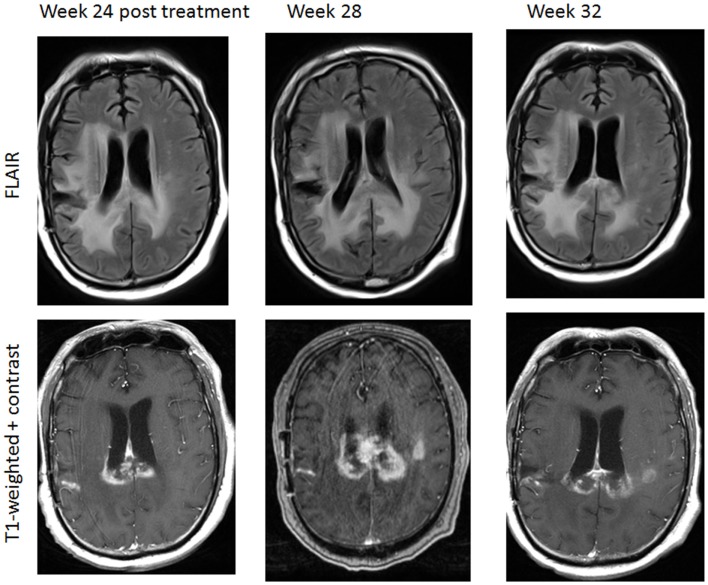
**A patient with progressive glioblastoma treated by immunotherapy including nivolumab (anti-PD1antibody) and ipilimumab (anti-CTLA-4 antibody)**. There was a transient increase in enhancing area in the posterior corpus callosum and left corona radiation 24 weeks following therapy initiation, consistent with pseudoprogression. Week 32 imaging was obtained after 1 week of corticosteroid therapy.

Ongoing immunotherapy trials for glioblastoma also show complex radiographic effects, including inflammation leading to the enlargement of pre-existing enhancing lesions or the appearance of new enhancing lesions (Figure 7). It is particularly challenging given current technologies to differentiate pseudoprogression caused by an anti-tumor mediated immune response from true progression using the RANO criteria, as timing of immune-mediated anti-tumor effects seems to differ from that seen with cytotoxic chemotherapies (173, 177, 178). The mechanism of contrast enhancement during pseudoprogression following immunotherapy is presently unclear and may be different from that following traditional chemoradiation therapy. Current efforts are ongoing to incorporate immune-related considerations into the RANO criteria (iRANO) to allow for improved response assessment and provide clinical guidelines for patients undergoing immunotherapy trials for glioblastoma. The iRANO criteria will define PD as persisting beyond a determined period of time after initial radiographic evidence of progression, allowing for patients with no significant neurological decline to stay on a potentially efficacious treatment longer and also allow for discontinuation of treatment earlier in the context of significant neurological decline. While iRANO criteria are currently being developed, advanced neuroimaging modalities useful in differentiating pseudoprogression from true progression may become invaluable in making clinical decisions in this rapidly evolving field of immunotherapy.

To date, only a few studies have evaluated advanced imaging techniques in characterizing pseudoprogression following immunotherapy. Vrabec et al. retrospectively assessed MR perfusion and diffusion imaging findings of eight patients with progressive/recurrent glioblastoma following dendritic cell immune therapy. In this small series, contrast-enhancing areas secondary to immune therapy-induced inflammation showed significant differences in maximum rCBV ratios and minimum ADC values compared to progressive/recurrent tumor (179). With a growing number of immunotherapy-based clinical trials for malignant glioma, the clinical and imaging characteristics of pseudoprogression with respect to each type of immunotherapy ultimately will become better understood. Most importantly, there is an urgent need to explore, validate, and standardize radiographic criteria based on both available and new imaging techniques for patients receiving immunotherapy in order to better define treatment efficacy.

## Conclusion

The updated criteria proposed by the RANO group have incorporated new guidelines to address the phenomena of pseudoprogression and pseudoresponse in patients with high-grade glioma. A minimum standard protocol necessary for evaluating radiographic response per RANO criteria should consist of pre- and post-contrast T1-weighted sequence as well as T2 and/or FLAIR sequences, ideally with the same magnetic field strength, acquisition parameters, and contrast dose throughout baseline and subsequent follow-up MRI studies to improve measurement reproducibility. The time interval between MRI studies immediately following radiation treatment is typically 1 month unless new clinical symptoms mandate earlier imaging. Advanced MRI sequences, such as perfusion and diffusion-weighted imaging, are also recommended as part of a standard imaging protocol to be readily evaluated in conjunction with conventional sequences when tumor progression or post-treatment changes are suspected. Other imaging techniques, such as MRS and PET, may help evaluate suspected lesion(s) and may require referral to centers with expertise in neuroimaging as well as neuro-oncology care.

With a growing number of new therapeutic options available for glioblastoma patients, diagnostic imaging tools allowing accurate characterizations of tumor response or resistance are urgently needed. We reviewed a number of advanced imaging methods for evaluating pseudoprogression following standard chemoradiation therapy and clinical trials including immunotherapy, as well as pseudoresponse in the setting of antiangiogenic therapy. As these techniques are increasingly incorporated into routine brain tumor imaging protocols, the sensitivity and specificity for detecting true tumor growth or shrinkage will be better defined. These advances should come with an emphasis on standardization and ease of implementation, which are required for subsequent validation and clinical use.

## Conflict of Interest Statement

The authors declare that the research was conducted in the absence of any commercial or financial relationships that could be construed as a potential conflict of interest.
